# Scale of Subtle Prejudices Towards Disability at the University: Validation in Mexican Population

**DOI:** 10.3390/ejihpe15040051

**Published:** 2025-04-01

**Authors:** Andrés Sánchez-Prada, Carmen Delgado-Álvarez, Alicia Gurdián-Fernández

**Affiliations:** 1 Facultad de Psicología, Universidad Pontificia de Salamanca, Compañía 5, 37002 Salamanca, Spain; mcdelgadoal@upsa.es; 2 Instituto de Investigación en Educación, Ciudad de la Investigación, Universidad de Costa Rica, San Pedro de Montes de Oca 11501-2060, Costa Rica; alicia.gurdian@ucr.ac.cr

**Keywords:** disability, higher education, evaluation, measurement scales, subtle prejudice

## Abstract

The purpose of this study was to analyze the adequacy of the “Subtle Prejudice Scale towards Disability in the University” for a university environment in Mexico. The theoretical model of this scale, which incorporates the gender dimension, was previously validated in Spain and Costa Rica with good psychometric properties and evidence of construct validity. The application of the scale in a Mexican university sample of 601 participants (83.4% students; 53.1% women and 45.3% men; aged 18 to 82, *M* = 25 years) confirmed the dimensional structure of the original four-factor model, with good fit indices through exploratory and confirmatory factor analysis and with adequate internal consistency for each dimension: avoidance of contact (ω = 0.81), benevolent idealization (ω = 0.77), excessive demands (ω = 0.73), and sexist amplification of prejudice (ω = 0.77). Relations between the scale dimensions and other variables (participants’ sex, political opinion, and preferred university policies for people with disabilities) were consistent with the current literature: men and right-wing people tend to show higher levels of ableism, which in turn are inversely associated with the supporting of inclusive policies. The results endorse the cultural validity of the scale and its suitability to evaluate ableism in Mexican universities.

## 1. Introduction

Discrimination is strongly linked to the stigma or social rejection experienced by individuals categorized as members of groups or collectives that do not conform to desirable social standards. As a result, the internalization of dominant ideological beliefs about these standards manifests in prejudiced attitudes and discriminatory behaviors toward individuals who embody the attributed characteristics of stigmatized groups (Quiles, 2019; Quiles & Morera, 2008).

The debate over appropriate terminology to refer to individuals whose corporeality does not align with normative standards has been a source of controversy, currently involving two different positions. On the one hand, the term “functional diversity” was proposed to describe corporeal differences without the stigma associated with deficiency or impairment (Romañach & Lobato, 2009). From this perspective, the stigmatization of women and men with functional diversity stems from their physical, sensory, or psychological divergence from the ideal corporeal model, leading to their perception and designation in terms of deficiency, impairment, or disability. On the other hand, some collectives of individuals affected by this functional diversity, such as the Spanish Committee of Representatives of Persons with Disabilities (CERMI, in the Spanish acronym), advocate for the continued use of the term “disability” to highlight that “this diversity” is not neutral but entails limitations that pose barriers in daily life. Proponents of this position reject terms like “functional diversity” or “different abilities”, arguing that they are paternalistic, “politically correct” euphemisms that obscure their real needs and, consequently, their right to have those needs met. The term “persons with disabilities”, adopted by the United Nations in 2006, is considered semantically correct by those defending this perspective (Comité Español de Representantes de Personas con Discapacidad, 2023). In this study, both terms will be used interchangeably, without implying a stance on the issue, preferably using “people with disabilities”, as affected people from some collectives ask to be named.

Prejudice and discrimination against individuals with some form of disability are well-documented phenomena across all societies, varying in intensity. In the case of Mexico, the most recent study conducted by the National Institute of Statistics and Geography (Instituto Nacional de Estadística y Geografía, 2022), in collaboration with the National Council to Prevent Discrimination (CONAPRED) and the National Human Rights Commission (CNDH), identified ten groups requiring visibility and recognition due to their historical discrimination. Among these groups are individuals with some form of disability, whose population rate in Mexico was estimated at 3.2%. According to the same study, 33.8% of individuals with disabilities reported having experienced some form of discrimination in the past 12 months, with 22.6% citing deprivation of opportunities to pursue or continue their education. For women, the double discrimination for being both disabled and female was reported by 23.7%, confirming that overlapping factors such as ethnicity, gender, or social class lead to multiple discrimination and amplify the experience of discrimination related to disability (Arellano et al., 2019).

Given that the deprivation or restriction of educational opportunities underpins other forms of discrimination, educational processes represent one of the most efficient mechanisms for fostering fair and equitable societies, making them a priority domain of action (Davis & Watson, 2001). However, advancing in the creation of inclusive educational communities that address the diverse needs of students requires identifying the obstacles and significant factors that can facilitate change (Fernández-Muñoz & Durán-Rojas, 2020). Since the right to inclusive higher education is enshrined in numerous national and international conventions and treaties, it is essential to recognize that this right is fulfilled only when the environment adapts to the specific needs of students with disabilities, rather than the other way around (Mareño Sempertegui, 2023; Palacios, 2019).

### 1.1. Prejudice Toward Disability in Higher Education

The term ableism emerged during the social movements in the United States and the United Kingdom in the 1960s to describe prejudice and discrimination against the community of people with disabilities (Brown, 2020). This term refers to a network of beliefs, processes, and practices that result in viewing disability as “a diminished state of being human” (Campbell, 2001, p. 44) due to its failure to conform to standard corporeality. The production of studies on ableism has increased in recent decades, driven by the awareness and activism of groups that have framed this issue within the realm of human rights. However, within the context of higher education, research on ableism remains scarce, and monographs on the topic have only recently begun to emerge. These include works published by the University of Michigan in English (e.g., Dolmage, 2017; Kerschbaum et al., 2017; Price, 2011) and a few publications in Spanish (e.g., Barradas, 2014; Cisternas et al., 2022; Norgazaray & Fraijo, 2022). Certainly, despite the lack of systematic reviews, in recent years greater attention has been directed toward research on disability in higher education, including Spanish-speaking contexts (Arellano et al., 2019; Fontana & Vargas, 2018; Fontana et al., 2015; Mareño-Sempertegui, 2021; Salinas, 2014; Suriá, 2017; Suriá et al., 2011). Nevertheless, a recent review of 31 quantitative studies on attitudes toward students with disabilities in higher education (Sainz et al., 2022) highlighted the paucity of empirical research that maps the current state-of-the-art to design effective university policies. This review also pointed to some noteworthy findings. For instance, the variable with the greatest influence on attitudes toward disability was personal contact with people with disabilities; individuals with little or no contact tended to believe that students with disabilities enjoyed unfair privileges and advantages. This finding had been reported in other studies, which showed that a higher frequency of contact correlated with more egalitarian attitudes toward disability (e.g., Fuentes et al., 2022; Garabal-Barbeira et al., 2017).

Regarding academic performance, the importance of the surrounding environment’s attitude had already been emphasized by demonstrating that the social climate of the campus, perceptions of self-efficacy, and personal lived experiences were the most predictive variables of graduation expectations (Fichten et al., 2014). The same study also revealed that women with disabilities, despite scoring higher than men on measures of self-efficacy, did not achieve better performance or graduation expectations as might be expected. This was attributed to their encountering less supportive personal situations and educational environments compared to men with disabilities, which nullified the positive effects of their higher self-efficacy perceptions. The attitudes of faculty and administrative staff also emerged as a factor influencing the performance of individuals with disabilities. In this sense, Sainz et al. (2022) highlighted the negative impact of perceiving students with disabilities as an additional workload and as a difficulty for which staff lack adequate training and resources.

Research on prejudice against people with disabilities, like any other form of prejudice, requires consideration of its various expressions, as demonstrated by the model of subtle prejudice in studies on racism (Pettigrew & Meertens, 2001). According to this model, subtle prejudice refers to a socially acceptable way of expressing prejudice without it being perceived as revealing bias (Meertens & Pettigrew, 1997). It takes the form of seemingly positive attitudes, yet it conceals the stigmatizing label that constructs the identity of “the different other”. This benevolent manifestation of ableism is expressed through pity, paternalism, or unwarranted praise (Nario-Redmond et al., 2019). In this sense, an analysis of the discourse of faculty members participating in a qualitative study on higher education (Vargas, 2013) identified the following characteristics of ableism: (a) it is not expressed explicitly and is unrecognized by those who exhibit prejudiced attitudes; (b) it has a strong emotional component manifested in responses of fear, contempt, rejection (hostile expression), or infantilizing paternalism and protectionism (benevolent expression); and (c) it is reflected in contradictions between practice and discourse.

These findings on ableism align with the principles of subtle prejudice theory in the context of racism (Pettigrew & Meertens, 2001) and ambivalent prejudice theory in the context of sexism (Glick & Fiske, 1996, 2001). The ambivalent sexism model identified a hostile dimension corresponding to traditional expressions of prejudice and a benevolent dimension tailored to cultural contexts where hostility is socially unacceptable. In the context of disability, the hostile dimension relates to a perception of deficit and impairment, while the benevolent dimension takes the form of condescending and paternalistic protection. In both cases, whether hostile or benevolent, functional diversity or specific disabilities become the defining elements of the individuals, subordinating them to their condition of impairment or deficiency, which remains the dominant aspect of their identity.

### 1.2. Measuring Attitudes and Prejudices Toward Disability

The publication of a monograph on measuring attitudes toward disability by the Human Resources Center (Yuker et al., 1970) constituted the first literature review aimed at developing a scale with evidence of reliability and validity, according to the psychometric standards of the time. Since then, it is estimated that approximately 25 self-report tools have been developed (Fuentes et al., 2022).

In Spanish-speaking countries, several instruments have been designed to measure these attitudes in the general population. One of the most popular tools in Spain and Latin America, due to its early availability, is the Scale of Attitudes toward Persons with Disabilities (EAPD in the Spanish acronym), developed in Spain by Verdugo et al. (1994). This 37-item Likert scale measures the appraisal of abilities and limitations, recognition and denial of rights, personal involvement, generalized rating, and role assumption. Psychometric adaptation for the Mexican population revealed a bifactorial structure, with one factor representing *equality* and the other representing *discrimination* (Sainz-Palafox et al., 2022).

Specifically in higher education, the more recent Questions about University and Disability Scale (CUNIDIS in the Spanish acronym; Rodríguez-Martín & Álvarez-Arregui, 2013, 2014) evaluates four dimensions, each with 10 Likert-type items: *curricular adaptations*, *teaching actions*, *accessibility*, and *university community*. As the scale’s name suggests, it focuses more on beliefs about how curricular adaptations and teaching practices should be implemented, as well as perceptions of accessibility and awareness within the university community, rather than being a direct measure of prejudice.

In the Latin American context, the Questionnaire on Attitudes towards Disability in Higher Education was also recently published (QAD-HE; Fuentes et al., 2022). This 27-item scale yields two factors through exploratory factor analysis: *egalitarian attitudes* (11 items) and *prejudiced attitudes* (16 items). Similar to the Mexican adaptation of the EAPD (Sainz-Palafox et al., 2022), the factorial solution of this scale does not represent two theoretical dimensions of prejudice but rather item clusters with differing theoretical content based on their favorable or unfavorable attitudinal valence.

Finally, the Scale of Subtle Prejudices towards Disability at the University (EPSDU, in the Spanish acronym; (Gurdián-Fernández et al., 2020a)), jointly developed in Costa Rica and Spain, is a 24-item instrument, with two introductory items aimed at encouraging sincere responses that do not contribute to the scale’s score. This scale measures prejudice toward disability in the university context through four dimensions: perceived *excessive demands* by the environment, *avoidance of contact* with people with disabilities, *benevolent idealization* of special traits in people with disabilities, and *sexist amplification* of prejudice. These indicators or dimensions are defined using the previously mentioned theoretical models: subtle prejudice theory (Pettigrew & Meertens, 2001), ambivalent sexism theory, with the benevolent dimension of prejudice (Glick & Fiske, 2001), and the multiple discrimination approach, understood as the combination of two or more factors that amplify prejudice (Crenshaw, 1991; Rey-Martínez, 2008). The suitability of these theoretical models for measuring ableism is supported by various empirical studies. For example, Nario-Redmond et al. (2019), based on the experiences of an international sample of 185 individuals with disabilities, reported the ambivalent conceptualization of prejudice toward people with disabilities and the multiple discrimination phenomenon. Specifically, the double discrimination faced by women with disabilities has also been documented in numerous studies (e.g., Davaki et al., 2013; De Silva, 2008; Martín-Cilleros et al., 2020; Mertens et al., 2007; Pineda & Luna, 2018; Timmons et al., 2024; Verdugo-Alonso et al., 2022).

From this theoretical framework, and based on published studies on the original version of the scale (Gurdián-Fernández et al., 2020a, 2020c), the four indicators of ableism were defined. Items for each indicator or dimension were drafted using verbatim statements collected from a university sample who had participated in a previous qualitative study (Vargas, 2013). Subsequently, experts rated the relevance and pertinence of the items for measuring the construct, resulting in the 24-item scale. This scale was then subjected to empirical validation in Costa Rica and Spain, with results described as “a promising starting point for future studies to gather additional validity evidence and test its utility for evaluation purposes” (Gurdián-Fernández et al., 2020b, p. 20).

Thus, considering the theoretical and technical characteristics of this instrument for assessing ableism in higher education, the aim of this study is to test the psychometric adequacy of the EPSDU for the Mexican university population by contrasting and expanding the range of cultural validity evidence (Solano-Flores & Nelson-Barber, 2001).

## 2. Materials and Methods

### 2.1. Participants and Procedure

The research protocol was approved by the Institutional Review Board of the University of Costa Rica (Ref. 724-C0-359) as part of an international research project in collaboration with other partner universities from Costa Rica, Spain, and Mexico (Ref. Pry01-2121-2020). For the current study, it was requested the collaboration of staff and students from the Mexican partner university. First, potential participants were informed about the study’s purpose and the free and voluntary nature of their eventual participation. It was clarified that their anonymous responses would be treated collectively and confidentially, becoming part of a dataset for statistical analysis, exclusively for the specified research purposes. Next, those who consented and agreed to participate completed the data gathering questionnaire in person and in a pencil-and-paper format. Completing the questionnaire required approximately 5 min, and participants received no compensation for their voluntary participation.

The incidental non-probabilistic sample consisted of 601 participants, with 272 (45.3%) men and 319 women (53.1%). Ages ranged from 18 to 82 years (*M* = 25.03, *SD* = 11.04), with no statistically significant age differences between men and women [*t* (585) = −0.103, *p* = 0.918]. Table 1 presents the basic descriptive characteristics of the sample.

### 2.2. Measures

The data collection questionnaire began with the Scale of Subtle Prejudices towards Disability at the University (EPSDU; Gurdián-Fernández et al., 2020a, 2020b). This scale consists of 24 items with a seven-point Likert response format, ranging from 1 (total disagreement) to 7 (total agreement), plus two initial items designed to encourage honest responses, which are not included in the scoring (see Appendix A). According to the original model by Gurdián-Fernández et al., the 24 items are distributed across four subscales, each containing six items: excessive demands (ED, α = 0.78), avoidance of contact (AC, α = 0.83), benevolent idealization (BI, α = 0.84), and sexist amplification (SA, α = 0.81). Higher scores indicate higher levels of prejudice toward individuals with disabilities.

The questionnaire also included items on sociodemographic variables used to describe the sample and a question regarding university policy: “I believe the most appropriate university policy for people with disabilities is...” with five ordered response categories: (1) Equal requirements and conditions as other students; (2) Following the same programs with adaptations to their specific disability; (3) Pursuing the same degrees but designed exclusively for students with disabilities; (4) Choosing other university-level education specifically designed for students with disabilities; and (5) Opting for other non-university education better adapted to their specific disability.

### 2.3. Data Analysis

In line with current measurement standards (American Educational Research Association et al., 2014), validity evidence based on the scale’s internal structure was analyzed by means of exploratory factor analysis (EFA) and confirmatory factor analysis (CFA). Both analyses followed a complete-cases approach (*n* = 549), and, according to Izquierdo et al. (2014), the sample was randomly divided into two halves: one subsample (*n* = 274) for the EFA, and the other (*n* = 275) for the CFA.

The EFA was conducted using FACTOR 10.8 (Lorenzo-Seva & Ferrando, 2006; Ferrando & Lorenzo-Seva, 2017), based on the polychoric correlations matrix due to the nature of the variables and the violation of the multivariate normality assumption. Consequently, the RULS method (Robust Unweighted Least Squares; Morata-Ramírez & Holgado-Tello, 2013; Yang-Wallentin et al., 2010) was employed and, assuming correlation between factors, Promax oblique rotation was applied. Factor selection followed a multi-criteria approach, analyzing the theoretical coherence between factors and items in combination with Parallel Analysis (Timmerman & Lorenzo-Seva, 2011) and comparative analysis of goodness-of-fit indices across resulting models. Robust indices were utilized: GFI (goodness of fit index), AGFI (adjusted goodness of fit index), CFI (comparative fit index), NNFI (non-normed fit index) and RMSEA (root mean square error of approximation).

Subsequently, the corresponding CFA was conducted with AMOS 23. The ULS (Unweighted Least Squares) method was applied for parameter estimation, a method shown to work well with Likert-type scales (Forero et al., 2009; Morata-Ramírez et al., 2015). For this confirmatory approach, we used as fit indices GFI, AGFI, NFI (normed fit index), and SRMR (standardized root mean square residual), as well as two parsimony indices based on the GFI and the NFI, respectively: PGFI and PNFI.

Based on the resulting factorial solution, reliability analyses were performed using Cronbach’s alpha and omega (McDonald, 1999), for the total scale and each subscale. Additionally, a repeated measures ANOVA compared scores across the subscales. Validity evidence based on relations to other variables (American Educational Research Association et al., 2014) was explored using the following tests: (a) MANOVA to examine possible differences by sex; (b) Kruskal–Wallis test to study the effect of political opinion; and (c) Spearman correlations to explore relationships between subscales’ scores and the opinion about the most appropriate university policies for people with disabilities. These analyses were carried out with SPSS 25.

## 3. Results

### 3.1. Validity Evidence Based on Internal Structure

#### 3.1.1. Exploratory Factor Analysis

The exploratory factor analysis (EFA), conducted with data from the first sample (*n* = 274), revealed an original structure composed of four correlated factors. The data were suitable for analysis (KMO = 0.857; *p* < 0.001 in the Bartlett sphericity test), and the factorial solution explained 61.84% of the variance. Table 2 presents the factor loadings from the rotated pattern matrix.

As shown in Table 2, the resulting factorial structure includes a first factor comprising the six items corresponding to the original avoidance of contact subscale (AC; Cronbach’s α = 0.79); a second factor made up of the six items from the benevolent idealization subscale (BI; α = 0.80); a third factor where the six items from the excessive demands subscale load (ED; α = 0.73); and a fourth factor composed of the six items from the sexist amplification subscale (SA; α = 0.77). The only item showing some ambiguity was Item 03 (“People with disabilities require too much help to get ahead”), which loaded 0.284 on Factor 3 (ED) and 0.312 on Factor 4 (SA). Although the loading was slightly higher on Factor 4, it was retained in Factor 3 according to the original model due to its conceptual coherence with the item’s content. The four factors showed moderate positive correlations with each other, ranging from 0.424 (Factor 1–Factor 4) to 0.554 (Factor 1–Factor 3), except between Factor 1 and 2, whose correlation was statistically null (0.110). Finally, regarding the scale’s reliability, the overall internal consistency was α = 0.875, with relatively lower but satisfactory indices for each subscale (Nunnally & Bernstein, 1994). Items’ corrected homogeneity indices (cHI) in their respective subscales were above 0.39, except for Item 03 (cHI = 0.247), which presented the aforementioned ambiguity.

In any case, the previous parallel analysis suggested two different factorial solutions, which were also explored by using the same extraction and rotation methods. On the one hand, a three-factor solution, based on the mean of the eigenvalue distribution obtained through simulation, explained 56.72% of the variance. This solution clearly defined the first two factors according to the respective items from the SA and BI subscales, while the third factor grouped the items from the ED and AC subscales. On the other hand, a two-factor solution, based on the 95th percentile of the eigenvalue distribution, explained 49.38% of the variance. This solution included a first factor combining items from the ED and AC subscales and a second factor comprising items from the SA and BI, although the structure was more ambiguous than the previous ones.

To compare the adequacy of the three solutions, we took the goodness-of-fit indices reported in Table 3 as a reference, following the criteria proposed by Hu and Bentler (1999) and Schermelleh-Engel et al. (2003). As shown, the most satisfactory fit was obtained with the initial four-factor model.

#### 3.1.2. Confirmatory Factor Analysis

Next, the models obtained through EFA were tested in the second sample (*n* = 275) through confirmatory factor analysis (CFA) to determine whether the results from the first analyses could be replicated. Once again, the criteria proposed by Hu and Bentler (1999) and Schermelleh-Engel et al. (2003) were used for absolute and incremental fit indices, while lower thresholds were assumed for parsimonious fit indices according to Mulaik et al. (1989). Table 4 presents the results obtained from this analysis.

This second analysis confirmed the inadequacy of the two-factor model, with fit indices for the three- and four-factor models being very similar, showing smaller differences than in the previous analyses. Given the similarity in goodness-of-fit between these two models, including the two parsimonious-fit indices, the original four-factor model was retained due to its theoretical coherence with the construct. Namely, this model distinguishes two aspects within the more hostile dimension of ableism (Gurdián-Fernández et al., 2020b): the perception of excessive demands, more cognitive in nature, and the avoidance of contact, more emotional in nature.

**Figure 1:**
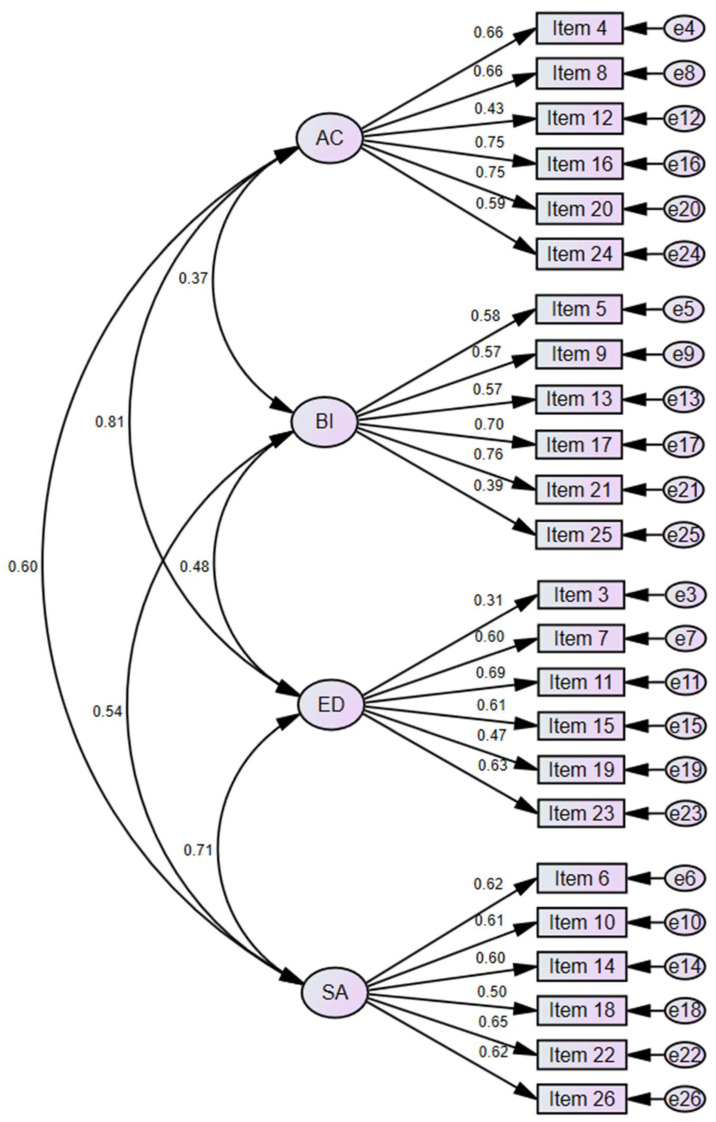
Four-factor model. AC = avoidance of contact; BI = benevolent idealization; ED = excessive demands; SA = sexist amplification.

Figure 1 displays the standardized regression coefficients and estimated correlations between factors. Factor loadings were acceptable for all items, with the lowest corresponding to Item 03. All factor correlations were positive and conceptually consistent. The highest correlation was observed between AC and ED factors (0.81), as expected given their more hostile nature compared to the other two factors. This was followed by the correlation between ED and SA factors (0.71), also theoretically expected due to their shared perception of “privilege” attributed to groups benefiting from compensatory measures against inequality. Lastly, the BI factor showed the lowest correlations with the other factors, particularly with AC (0.37), which is consistent with the theoretical model.

Considering both alpha and omega coefficients, internal consistency was satisfactory for every subscale (Cheung et al., 2024) in this subsample: AC (α = 0.80; ω = 0.81); BI (α = 0.76; ω = 0.77); ED (α = 0.71; ω = 0.73); and SA (α = 0.76; ω = 0.77). However, the respective average variances extracted (AVEs) did not achieve the heuristic 0.50 cut-off threshold proposed by Fornell and Larcker (1981) for *convergent validity*. We also applied the Fornell–Larcker AVE/SV approach to *discriminant validity* by comparing the AVE of each pair of constructs versus the shared variance (SV) between them. In this sense, and according to the observed correlations between factors, the AVE > SV criterion was achieved only for the BI subscale, while AC and SA showed certain overlaps with ED. These results are displayed in Table 5.

#### 3.1.3. Comparison of Scores Across Factors

The repeated measures ANOVA, after applying the Greenhouse–Geisser correction, detected significant differences among the factors, *F* (2.519, 1380.428) = 706.962, *p* < 0.001, η^2^ = 0.0563. Pairwise comparisons, adjusted using Bonferroni correction, revealed significant differences across the four subscales: the highest level of prejudice was observed in the BI dimension (*M* = 3.82, *SD* = 1.28), followed by ED (*M* = 2.61, *SD* = 1.06), then SA (*M* = 2.13, *SD* = 1.03), and finally, the lowest scores were observed in AC (*M* = 1.61, *SD* = 0.91).

### 3.2. Validity Evidence Based on Relations to Other Variables

#### 3.2.1. Relations to Sex and Political Opinion

**Figure 2:**
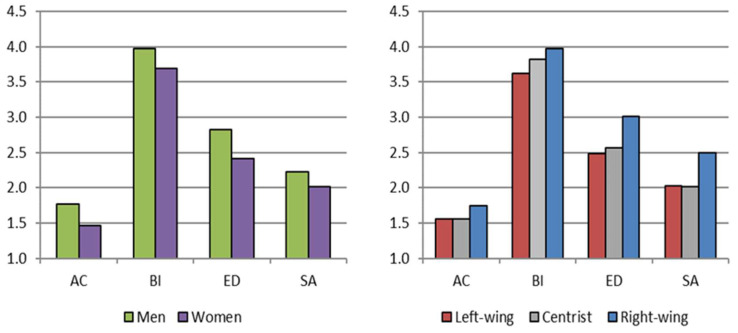
Differences by sex and political opinion. AC = avoidance of contact; BI = benevolent idealization; ED = excessive demands; SA = sexist amplification.

The MANOVA yielded a significant effect of sex on the multivariate vector (Pillai’s Trace: *F* (4, 534) = 6.511; *p* < 0.001; η^2^ = 0.047). Significant differences were observed across all prejudice dimensions, with men exhibiting higher levels of prejudice (see Table 6, Figure 2). The greatest differences between men and women were found in the two most hostile dimensions of prejudice, ED and AC, with effect sizes of 3.9% and 2.9%, respectively. Since effects of sex on BI and SA appeared to be small (Cohen, 1988), these relatively stronger effects are, in any case, modest in size.

Regarding political opinion, given the unequal group sizes, the non-parametric Kruskal–Wallis test was employed. A significant effect was found in two dimensions (ED and SA), and, as shown in Table 6 and Figure 2, the effect was the same in both cases: individuals identifying with right-wing ideologies scored higher in ED (*p* < 0.001) and in SA (*p* ≤ 0.002) compared to those identifying with centrist or left-wing ideologies. There were no significant differences between the latter two groups (*p* = 0.425 in ED; *p* = 0.913 in SA).

#### 3.2.2. Relations to the Opinion About University Policies for People with Disabilities

Finally, correlations between prejudice scores and opinions on the most appropriate university policy for students with disabilities were estimated. As shown in Table 7, the ED and AC subscales, which correspond to the most hostile dimension of prejudice, showed positive, albeit low, correlations. Consistent with expectations, this indicates that higher levels of ableism are associated with a greater tendency to support exclusionary policies, especially in the case of ED. Additionally, a positive correlation was found with the BI subscale, while no correlation was observed with SA.

## 4. Discussion

This study analyzed the construct validity of the Scale of Subtle Prejudices towards Disability at the University (EPSDU; Gurdián-Fernández et al., 2020a, 2020b) within a Mexican university context. Prejudice against disability in educational settings constitutes an obstacle to achieving equity and respecting human rights. Experimental studies have shown that individuals with disabilities are often perceived as less competent, which negatively impacts their expectations and performance (Rohmer & Louvet, 2018). However, adopting inclusion strategies can provoke hostile reactions and rejection of positive action measures as unjustified or unfair unless prejudice is addressed (Nario-Redmond & Oleson, 2016; Sherry, 2016). Having instruments that evaluate prejudice and identify specific dimensions for intervention design is therefore essential for implementing inclusive policies in higher education. From this perspective, considering current psychometric standards (American Educational Research Association et al., 2014), theoretical grounding and technical development make the EPSDU a promising tool for assessing subtle ableism. The results obtained in this study represent an initial, albeit sound, endorsement for its eventual application to Mexican university populations.

The EFA reproduced the four-factor structure as defined in the scale’s theoretical model, supporting its cultural validity (Solano-Flores & Milbourn, 2016; Solano-Flores & Nelson-Barber, 2001). This result was confirmed by the CFA, which yielded good fit indices and reliability coefficients. The theoretical foundation of the four-factor model provided an advantage over alternative two- and three-factor models that were also tested. In fact, if we take into account exclusively statistical arguments, both the three- and four-factor models resulted in similar goodness-of-fit and parsimony indices. It is the conceptual and practical relevance of distinguishing two latent variables within the hostile dimension of ableism that emerged as the main criterion to opt for the four-factor solution. In this sense, the multidimensional structure of the EPSDU includes an evaluative-cognitive component, the perception of excessive demands, which reflects negative evaluations of functional diversity as a deficit or deviation from the norm, resulting in reduced recognition of rights (Rodríguez & Ferreira, 2010). In addition, the avoidance of contact represents an evaluative–affective component, reported in previous studies as the most significant aspect of ableist attitudes (Fuentes et al., 2022; Garabal-Barbeira et al., 2017; Sainz et al., 2022). These two dimensions were highly correlated in the present study, confirming the close relationship between the beliefs underpinning prejudice and its emotional nature. Promoting collaborative activities between individuals with and without disabilities could effectively address both components, as greater frequency of contact and personal involvement is associated with more egalitarian attitudes toward disability (Fuentes et al., 2022; Garabal-Barbeira et al., 2017).

Recalling the very nature of subtle ableism, benevolent idealization, a socially acceptable form of expressing prejudice, yielded the highest scores. This is consistent with studies indicating that this is the predominant form of prejudice in societies committed to human rights and values of justice and equity (Fiske, 2001). Aligning with current research on modern ableism (e.g., Delgado-Álvarez et al., 2020; Friedman, 2019, 2024; Nario-Redmond et al., 2019), “more explicit” forms of prejudice measured through self-report scales tend to be underreported due to social desirability bias, as one characteristic of prejudice is its denial. Therefore, the relevance of such studies lies not in absolute scores but in relative scores that highlight the dimensions with the highest values, guiding the design of targeted interventions.

The fourth dimension, namely sexist amplification, represents an innovative contribution to measuring ableism by incorporating the interaction with gender, as recommended by European Parliament reports (Davaki et al., 2013). This dimension was most strongly correlated with the cognitive dimension of excessive demands, consistent with results from the original studies in Spain and Costa Rica (Gurdián-Fernández et al., 2020b), indicating that both factors share a perception of unjustified privilege in positive action efforts. These efforts, aimed at compensating structural inequality in access to universal rights, are often not accompanied by the necessary awareness and training actions to foster understanding (Nario-Redmond & Oleson, 2016; Verdugo-Alonso et al., 2022).

Beyond theoretical grounds, empirical validity criteria proposed by Fornell and Larcker (1981) were not completely accomplished. As previously mentioned, the four factors’ AVEs did not achieve the ideal 50% threshold, i.e., factor loadings above 0.70, although each construct explained more than 25% of the variance of their respective indicators, i.e., factor loadings above 0.50 (Hair et al., 2010; Cheung et al., 2024), at least considering average estimates. Likewise, the Fornell–Larcker criterion for considering factors’ discriminant capacity was met only for the BI dimension. Setting current criticisms about the “conservativeness” of the AVE/SV rule (e.g., Cheung et al., 2024; Rönkkö & Cho, 2022) aside, this finding should not be considered a problem on its own, since moderate-high correlations between dimensions were expected according to the constructs nomological net (Messick, 1998; Shepard, 1997). This implies that such dimensions are defined in part by their interrelations and therefore, according to Henseler et al. (2015, p. 131), “hindsight failure to establish discriminant validity between two constructs does not necessarily imply that the underlying concepts are identical”. On the contrary, in this case one strength of the EPSDU would be that it provides some nuances in the construct’s definition. Its multidimensional complexity could eventually allow for more targeted interventions.

These foundations of construct validity on both empirical evidence and theoretical congruence (Henseler et al., 2015; Messick, 1989) become manifest regarding decision-making on item 3. This item cross-loaded on the ED and SA factors in the EFA and showed the weakest loading on its assigned factor in the subsequent CFA. However, it was retained in the ED factor based on theory-driven arguments and not only on statistical outcomes. In any case, this item should be revised in further applications of the EPSDU; maybe it is a candidate to be dropped due to construct irrelevance issues (American Educational Research Association et al., 2014), or maybe it is a matter of wording and content ambiguity in the specific Mexican context: could the word “demasiado” [“too much”] have led to confusion? What did “too much” exactly mean for participants? And for whom was it “too much help”, for the helping person or for the person with a disability? All of these speculations might be working hypotheses for future studies aimed at shedding light on the structural and functional robustness of the EPSDU. At the moment, we consider that empirical evidence is not sufficient for making a decision against theoretical criteria, taking into account the good item’s adequacy found in previous research with the original version of the scale (Gurdián-Fernández et al., 2020b) and given that this study constitutes a first application to the Mexican population.

Keeping the above in mind, relations between the ESPDU scores and other variables are consistent with the literature on the subject. The differences found between men and women in all of the four dimensions of ableism reflect higher levels of prejudice in the male population, especially in its more hostile expression (Glick & Fiske, 1996; Glick et al., 2000; Salinas, 2014; Sainz-Palafox et al., 2022; Suriá et al., 2011). Likewise, the relation between political opinion and ableism was in the expected sense; more conservative leanings appear linked to more hostile (ED) and sexist (SA) forms of prejudice (Austin & Jackson, 2019; Christopher & Mull, 2006; Friedman, 2019; Frederick, 2019). Finally, a coherent tendency was also observed in relation to the preferred university policies for students with disabilities. The association found with the two hostile and the benevolent dimensions of the EPSDU underscores the role of positive attitudes, as opposed to ableism, as a facilitating factor for generating more flexible, respectful, and inclusive environments (Roca-Hurtuna et al., 2021; Sainz et al., 2022; Salinas, 2014).

## 5. Conclusions

The results obtained in this first study on the applicability of the EPSDU to the measurement of ableism in the Mexican university environment are promising. Nonetheless, this study has limitations that should be considered in future applications.

First, the sample was extracted from a single, public university, primarily composed of health science students, which makes necessary replication with more diverse samples across public and private institutions and across academic disciplines and university roles. In this sense, the lack of homogeneity in the sample sizes has prevented the present study from making comparisons by field of knowledge or according to participants’ positions at the university, which might yield interesting insights. New studies providing evidence from students and both academic and non-academic staff, with larger and more balanced samples, are warranted. From a psychometric standpoint, it will contribute to reinforcing validity evidence based on the scale’s internal structure and response processes (American Educational Research Association et al., 2014), for example, by performing measurement invariance tests across groups of interest. Such studies will allow for a more nuanced understanding of one of the main social stressors that affect people with disabilities, filling a gap in the current literature (Wolbring & Escobedo, 2023).

Second, future studies should incorporate additional measures of constructs that are convergent or divergent with subtle ableism to broaden the range of construct validity evidence. For example, the EPSDU could be applied together with previously validated measures of attitudes such as the EAPD (Verdugo et al., 1994; Sainz-Palafox et al., 2022) or the QAD-HE (Fuentes et al., 2022), as well as with other partially related instruments like the Ambivalent Sexism Inventory (Glick & Fiske, 1996). Another variable that would be interesting to include along with the EPSDU is social desirability, since the instrument is not immune to its effects. Therefore, it would be important to include a measure of social desirability in future studies to assess and potentially control its impact on ableism scores, thereby optimizing the evaluation process. Concretely, procedures like those proposed by Ferrando et al. (2009) would be useful for item calibration and estimation of the EPSDU factor scores once removed the variance explained by social desirability. A posteriori control of such a confounding variable could also be possible by means of ANCOVA. Finally, it would be interesting to include other factors that interact with ableism beyond gender, which would contribute to increasing the amount of explained variance in a broader multidimensional framework, such as, for example, socioeconomic status, ethnicity, or race (e.g., Mireles, 2022). In line with this last study, another interesting future direction could consist in incorporating the qualitative approach along with self-report measures like the EPSDU in order to deepen into the experience of people with disabilities at the university and “to bring invisible voices to the research agendas” (Lillywhite & Wolbring, 2022, p. 17).

In summary, despite all the limitations and opened issues, the statistical robustness and conceptual coherence of the results obtained in this study provide evidence of the scale’s psychometric adequacy for assessing prejudice toward disability in Mexican higher education. It could help to identify subtle ableist narratives that maintain the “othering” discourse (Wolbring & Lillywhite, 2021), as well as specific needs that, ultimately, could contribute to strengthening equity/equality, diversity, and inclusion (EDI) policies for people with disabilities at the university (Brown & Leigh, 2020). In this sense, once the above-mentioned limitations are addressed and reinforce the validity argument for using the EPSDU (American Educational Research Association et al., 2014; Kane, 1992, 2001), several practical implications might emerge. First, future findings could contribute to guiding policies or interventions based on robust evidence, which would permit a more fine-grained understanding of one of the main psychosocial barriers that people with disabilities face in their everyday experience (Gurdián-Fernández et al., 2020a; Wolbring & Escobedo, 2023). Second, such evidence-based understanding could lead to a critical self-questioning of the subtle, rationalized, and somehow socially tolerated manifestations of ableism that hinder the implementation of effective EDI policies (Wolbring & Lillywhite, 2021). Third, the EPSDU could contribute to this self-questioning by informing new public campaigns aimed at increasing awareness among university communities, as well as specific interventions targeted at prominent dimensions of prejudice. For instance, once it was confirmed that benevolent idealization is currently one of the most predominant forms of prejudice, as expected in egalitarian societies (Fiske, 2001; Nario-Redmond et al., 2019), the need for “deconstructing” the benevolence discourse would become a priority in order to show the negative subtract (and consequences for people with disabilities) of this apparently well-meaning narrative. This deconstructive approach might also work against other internalized myths, stereotypes, and rationalized-but-spurious arguments like those related to the excessive demands and sexist amplification dimensions (Mareño Sempertegui, 2023; Verdugo-Alonso et al., 2022). Finally, regarding avoidance of contact, its emotional nature might make it necessary to combine that self-reflective approach with some more experiential ones, as, for example, the promotion of contact and collaboration between people with and without disabilities in their daily life (Fuentes et al., 2022; Garabal-Barbeira et al., 2017). These could be, in our opinion, some of the eventual contributions of the EPSDU to the development and consolidation of a more inclusive higher education.

## Figures and Tables

**Table 1:** Descriptive characteristics of the sample.

Variable	Category	*n* (%)
Sex	Men	272 (45.3%)
	Women	319 (53.1%)
	No response	10 (1.6%)
Position at university	Student	501 (83.4%)
	Academic staff	47 (7.9%)
	Administrative staff	28 (4.7%)
	Other	11 (1.8%)
	No response	14 (2.2%)
Field of knowledge	Health Sciences	447 (74.4%)
	Economics—Administration	72 (12.0%)
	Technics	61 (10.1%)
	Arts	21 (3.5%)
Political opinion	Right-wing	104 (17.3%)
	Centrist	367 (61.1%)
	Left-wing	78 (13.0%)
	No response	52 (8.6%)

**Table 2:** Rotated loadings matrix. Four-factor solution.

Item (Subscale) ^1^	Factor 1	Factor 2	Factor 3	Factor 4
Item 16 (AC)	**0.836**	−0.059	−0.016	0.087
Item 04 (AC)	**0.818**	0.083	−0.154	−0.088
Item 20 (AC)	**0.790**	0.025	0.047	0.094
Item 12 (AC)	**0.778**	−0.111	0.015	0.037
Item 08 (AC)	**0.697**	−0.062	0.104	0.023
Item 24 (AC)	**0.677**	0.045	0.092	0.122
Item 09 (BI)	0.181	**0.811**	−0.007	−0.160
Item 17 (BI)	−0.013	**0.749**	−0.106	0.135
Item 13 (BI)	−0.059	**0.730**	−0.044	0.150
Item 21 (BI)	0.000	**0.653**	0.098	0.020
Item 25 (BI)	−0.254	**0.590**	0.131	0.048
Item 05 (BI)	0.002	**0.435**	0.175	0.113
Item 23 (ED)	0.066	0.101	**0.788**	−0.101
Item 11 (ED)	−0.152	−0.100	**0.687**	0.346
Item 15 (ED)	0.282	0.165	**0.582**	−0.253
Item 07 (ED)	0.316	0.014	**0.504**	−0.010
Item 19 (ED)	0.210	−0.089	**0.452**	0.002
Item 03 (ED)	−0.155	0.034	**0.284**	0.312
Item 14 (SA)	0.059	−0.067	−0.038	**0.868**
Item 26 (SA)	0.098	−0.093	0.142	**0.677**
Item 10 (SA)	0.023	0.139	−0.064	**0.666**
Item 22 (SA)	0.090	0.058	0.058	**0.660**
Item 06 (SA)	0.067	0.181	−0.225	**0.642**
Item 18 (SA)	0.000	0.101	0.149	**0.432**

^1^ Theoretical subscales according to Gurdián-Fernández et al. (2020b) model: AC = avoidance of contact; BI = benevolent idealization; ED = excessive demands; SA = sexist amplification. Loadings corresponding to the assigned factors are presented in bold font.

**Table 3:** Results of exploratory factor analysis (RULS). GOF indices.

Fit Index	Good/Acceptable Fit	4 Factors	3 Factors	2 Factors
GFI	≥0.95/≥0.90	0.989	0.983	0.967
AGFI	≥0.90/≥0.85	0.983	0.977	0.960
CFI	≥0.97/≥0.95	0.997	0.987	0.961
NNFI	≥0.97/≥0.95	0.995	0.983	0.953
RMSEA [95% CI] ^1^	≤0.05/≤0.08	0.024 [0.038, 0.072]	0.045 [0.053, 0.088]	0.074 [0.077, 0.109]

^1^ Confidence intervals were estimated based on 500 bootstrap samples.

**Table 4:** Results of confirmatory factor analysis (ULS). GOF indices.

Fit Index	Good/Acceptable Fit	4 Factors	3 Factors	2 Factors
GFI	≥0.95/≥0.90	0.953	0.951	0.928
AGFI	≥0.90/≥0.85	0.943	0.941	0.914
PGFI	≥0.50	0.782	0.789	0.776
NFI	≥0.95/≥0.90	0.920	0.916	0.876
PNFI	≥0.50	0.820	0.826	0.797
SRMR	≤0.05/≤0.08	0.067	0.070	0.083

**Table 5:** AVEs and shared variances between constructs.

Factor	AC	BI	ED	SA
AC	**0.423**	0.134	0.664	0.358
BI	0.134	**0.367**	0.230	0.287
ED	0.664	0.230	**0.323**	0.498
SA	0.358	0.287	0.498	**0.363**

Note: Diagonal values in bold font correspond to the average variance extracted (AVE) for each construct. Squared correlations involving the constructs (i.e., shared variance) are presented outside the diagonal. AC = avoidance of contact; BI = benevolent idealization; ED = excessive demands; SA = sexist amplification.

**Table 6:** Differences between groups on prejudice dimensions.

	Subscale	Group’s Mean (SD)	Effect
Sex	Avoidance of Contact (AC)	Men: 1.77 (1.03) Women: 1.46 (0.73)	*F* (1, 537) = 16.092; *p* < 0.001; η^2^ = 0.029
	Benevolent Idealization (BI)	Men: 3.97 (1.29) Women: 3.69 (1.26)	*F* (1, 537) = 6.071; *p* = 0.014; η^2^ = 0.011
	Excessive Demands (ED)	Men: 2.82 (1.14) Women: 2.41 (0.92)	*F* (1, 537) = 21.988; *p* < 0.001; η^2^ = 0.039
	Sexist Amplification (SA)	Men: 2.22 (1.11) Women: 2.02 (0.93)	*F* (1, 537) = 5.286; *p* = 0.022; η^2^ = 0.010
Political opinion	Avoidance of Contact (AC)	Right-wing: 1.75 (1.08) Centrist: 1.56 (0.83) Left-wing: 1.56 (0.85)	*H* (2) = 1.990; *p* = 0.370
	Benevolent Idealization (BI)	Right-wing: 3.97 (1.22) Centrist: 3.82 (1.30) Left-wing: 3.62 (1.28)	*H* (2) = 2.362; *p* = 0.307
	Excessive Demands (ED)	Right-wing: 3.01 (1.11) Centrist: 2.56 (1.02) Left-wing: 2.48 (1.10)	*H* (2) = 16.927; *p* < 0.001
	Sexist Amplification (SA)	Right-wing: 2.49 (1.06) Centrist: 2.02 (0.95) Left-wing: 2.03 (0.96)	*H* (2) = 18.365; *p* < 0.001

**Table 7:** Correlations with opinions on university policies.

	Avoidance of Contact	Benevolent Idealization	Excessive Demands	Sexist Amplification
Opinion about the most appropriate policies	0.113 **	0.124 **	0.152 ***	0.068

** *p* < 0.01; *** *p* < 0.001.

## Data Availability

Raw data supporting the reported results are available from the corresponding author upon request.

## Appendix A

Original (Spanish) version of the “Escala de Prejuicios Sutiles hacia la Discapacidad en la Universidad” (EPSDU) [Scale of Subtle Prejudices towards Disability at the University] as applied in the present study.

	**1 = Total DESACUERDO** **7 = Total ACUERDO**							
01 La libertad de expresión es un valor; por tanto debería ser políticamente correcto expresar cualquier opinión siempre que se haga respetuosamente.		1	2	3	4	5	6	7
02 No debería ser un tabú hablar sobre la discapacidad; las personas deberían expresar libremente lo que piensan sobre este tema, como sobre cualquier otro.		1	2	3	4	5	6	7
03 A las personas con discapacidad hay que ayudarlas demasiado para que salgan adelante.		1	2	3	4	5	6	7
04 En general me incomoda interactuar con una persona con discapacidad.		1	2	3	4	5	6	7
05 Las personas con discapacidad, en comparación con las demás personas, tienden a ser más compasivas por su discapacidad.		1	2	3	4	5	6	7
06 Los estudiantes con discapacidad participan más en las clases que las estudiantes con discapacidad porque ellas son calladas y tímidas.		1	2	3	4	5	6	7
07 La ayuda que requieren las personas con discapacidad para ingresar al mundo laboral implica una carga para las demás personas.		1	2	3	4	5	6	7
08 Aunque no discrimino a las personas con discapacidad, he de reconocer que me intimida relacionarme con ellas.		1	2	3	4	5	6	7
09 Las personas con discapacidad tienen unos valores que pocas personas suelen tener, precisamente por la vivencia de su discapacidad.		1	2	3	4	5	6	7
10 Los estudiantes con discapacidad socializan más y mejor en el ambiente universitario que las estudiantes con discapacidad.		1	2	3	4	5	6	7
11 Las personas con discapacidad no pueden valerse por sí mismas, siempre requieren ayuda de los demás.		1	2	3	4	5	6	7
12 Me asusta la idea de tener que interactuar con una o un estudiante con discapacidad.		1	2	3	4	5	6	7
13 La mayoría de las personas con discapacidad se caracterizan por una simpatía que pocas personas poseen.		1	2	3	4	5	6	7
14 Los estudiantes con discapacidad pueden cursar más fácilmente carreras de tecnología y ciencias que las estudiantes con discapacidad, porque las mujeres tienen menor capacidad de abstracción.		1	2	3	4	5	6	7
15 Es absurdo negar que las personas con discapacidad son un problema, pues es evidente que tienen limitaciones.		1	2	3	4	5	6	7
16 Sinceramente, prefiero no relacionarme con personas con discapacidad.		1	2	3	4	5	6	7
17 Las personas con discapacidad, en comparación con las que no tienen ningún tipo de discapacidad, tienden a tener mayor sensibilidad moral.		1	2	3	4	5	6	7
18 Probablemente, las mujeres sufren la discapacidad más que los hombres pues para ellas es más importante su atractivo físico.		1	2	3	4	5	6	7
19 Tener una discapacidad debe ser como tener una enfermedad.		1	2	3	4	5	6	7
20 A pesar de que debemos respetar a las demás personas, siento incomodidad cuando hay un o una estudiante con discapacidad en mi aula.		1	2	3	4	5	6	7
21 Las personas con discapacidad tienen una sensibilidad mayor que las demás, por la limitación que implica su discapacidad.		1	2	3	4	5	6	7
22 Los recursos de la universidad son mejor aprovechados por los estudiantes con discapacidad que por las estudiantes con discapacidad, pues ellos tienen mejor rendimiento académico.		1	2	3	4	5	6	7
23 Las personas con discapacidad no son autónomas; por eso demandan más ayuda.		1	2	3	4	5	6	7
24 Si una persona acompaña a una persona con discapacidad prefiero comunicarme con ella que con la persona con discapacidad.		1	2	3	4	5	6	7
25 Las personas con discapacidad deberían ser admiradas por lo que les implica vivir con una limitación.		1	2	3	4	5	6	7
26 Para el personal docente es más problemático tener estudiantes mujeres con discapacidad pues tienen menor motivación para los estudios que los estudiantes con discapacidad.		1	2	3	4	5	6	7
	Dimensions—Subscales: “Exceso de demandas” [excessive demands (ED)] = items 3, 7, 11, 15, 19, and 23; “Evitación de contacto” [avoidance of contact (AC)] = items 4, 8, 12, 16, 20, and 24; “Idealización benevolente” [benevolent idealization (BI)] = items 5, 9, 13, 17, 21, and 25; “Amplificación sexista” [sexist amplification (SA)] = items 6, 10, 14, 18, 22, and 26.							
